# Current Knowledge about CD3^+^CD20^+^ T Cells in Patients with Multiple Sclerosis

**DOI:** 10.3390/ijms25168987

**Published:** 2024-08-18

**Authors:** Borros Arneth

**Affiliations:** 1 Institute of Laboratory Medicine and Pathobiochemistry, Molecular Diagnostics, Hospital of the Universities of Giessen and Marburg (UKGM), Justus Liebig University Giessen, Feulgenstr. 12, 35392 Giessen, Germany; borros.arneth@staff.uni-marburg.de; 2 Institute of Laboratory Medicine and Pathobiochemistry, Molecular Diagnostics, Hospital of the Universities of Giessen and Marburg (UKGM), Philipps University Marburg, Baldinger Str., 35043 Marburg, Germany

**Keywords:** CD3^+^CD20^+^ T cells, CD20-expressing, rituximab, multiple sclerosis, CNS, pathogenesis

## Abstract

Multiple sclerosis (MS) is a disease of the central nervous system (CNS) characterized by inflammation and autoimmune responses. This review explores the participation of T cells, particularly certain CD3^+^CD20^+^ T cells, in the clinical manifestations of MS and highlights their presence in diagnosed patients. These T cells show aberrant expression of CD20, normally considered a B-cell marker. In this review, relevant journal articles available in PubMed and CINAHL were identified by employing diverse search terms, such as MS, CD3^+^CD20^+^ T cells, the incidence and significance of CD3^+^CD20^+^ T cells in MS patients, and the impact of rituximab treatment. The search was limited to articles published in the ten-year period from 2014 to 2024. The results of this review suggest that most scholars agree on the presence of CD3^+^CD20^+^ T cells in cerebrospinal fluid. Emerging concepts relate to the fundamental role of CD20-expressing T cells in determining the target and efficacy of MS therapeutics and the presence of T cells in the cerebrospinal fluid of MS patients. The results clearly show that CD20^+^ T cells indicate disease chronicity and high disease activity.

## 1. Introduction

Multiple sclerosis (MS) is a disease of the spinal cord and brain that manifests as inflammation characterized by localized lymphocyte infiltration, which damages axons. Both B and T cells play roles in the pathogenesis of MS. Although the precise pathophysiological mechanism of MS remains incompletely understood, recent investigations have indicated that MS is predominantly influenced by autoreactive T cells [1]. Specifically, CD3^+^CD20^+^ T cells have been detected in the peripheral blood and cerebrospinal fluid (CSF) of patients diagnosed with MS [2]. Furthermore, this subpopulation of cells can be effectively depleted in rituximab-treated MS patients. A decrease in the T-cell population is observed in MS. CD3^+^CD20^+^ T cells can be considered the population of CD3^+^ T cells that co-express CD20; these cells account for approximately 5% of the CD3^+^ compartment in the peripheral blood of humans [3].

CD20-expressing T cells (CD3-positive cells) constitute a very unusual cell population that is not normally found in healthy people. CD20 is normally a B-cell epitope or marker that is not routinely found on T cells. However, on the specific cells investigated here from patients with MS, the CD20 marker is expressed on CD3^+^ T cells, which can be interpreted as aberrant receptor expression in the context of a chronic disease. According to the literature reviewed herein, CD20^+^CD3^+^ T cells are found in increased numbers in some patients with MS. Since this is a very unusual phenomenon, the following review aimed to compile recent and current literature on this phenomenon.

Earlier research showed the pathogenic tendencies of CD3^+^CD20^+^ T cells in autoimmune disorders and CD4^+^ T-cell malignancies [4]. From an immunological perspective, the putative B-cell antigen CD20 is expressed on the surface of B cells, as well as on late pro-B and memory cells, but not early pro-B cells. Therefore, CD20 is considered a lymphatic B-cell marker, whereas CD3 is a T-cell marker [5,6]. Currently, ample evidence indicates that CD20 is expressed in a subset of CD3^+^ T cells within the peripheral blood of healthy individuals and those diagnosed with cancer or autoimmune diseases such as MS [7]. Consequently, this specific subpopulation is categorized as CD20-expressing CD3^+^ T cells (CD3^+^CD20^+^ T cells). Recent studies have confirmed that CD3^+^CD20^+^ T cells display similar but unique biological characteristics compared to conventional CD3^+^ T cells [8], and thus play a critical role in human disease. Hence, this systematic review clearly outlines the role and presence of CD3^+^CD20^+^ T cells in MS patients.

## 2. Materials and Methods

**Figure 1:**
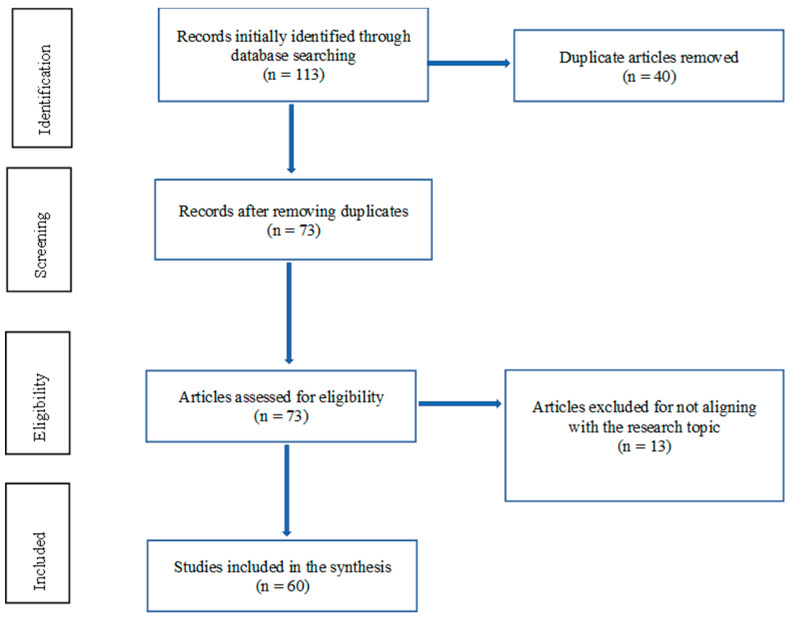
The process by which each study was considered for inclusion in the systematic review and the factors for exclusion. In the first stage, 113 studies were identified for further assessment. The second phase involved screening for duplicates: 40 studies were eliminated, leaving 73 studies for further evaluation. The third phase involved checking for study eligibility: 13 studies were thus excluded for various reasons, such as not meeting the inclusion and exclusion criteria. In this third phase, the greatest consideration was given to study alignment with the research objective of addressing the occurrence and role of CD3^+^CD20^+^ T cells in MS. Finally, only 60 studies were included in the systematic review.

This systematic review was performed in accordance with the Preferred Reporting Items for Systematic Reviews and Meta-Analyses (PRISMA) reporting guideline. The PRISMA flow chart of this study is given in Figure 1.

### 2.1. Patient Involvement

Public or patient participation was not considered when designing or conceptualizing the research for this systematic review.

### 2.2. Identification of Studies

Studies were identified by targeted literature searches of the Embase, PubMed, and ResearchGate databases. The primary sources considered were original peer-reviewed research articles, conference presentations, expert opinion articles, and review articles. The literature databases were searched using key terms such as CD3^+^CD20^+^ T cells, multiple sclerosis, rituximab, the role of T cells in multiple sclerosis, CD20-expressing T cells, and the occurrence of CD3^+^CD20^+^ T cells. The search strategy prioritized studies published only in English. Advanced search filters were used to retrieve experimental articles, specifically those on patients diagnosed with MS.

### 2.3. Inclusion and Exclusion Criteria

The studies considered for inclusion in this review focused on humans and were conducted in various settings, including community and clinical facilities encompassing primary, secondary, and tertiary centers. To meet the search criteria, studies were also required to be relevant to the research topic of the occurrence and role of CD3^+^CD20^+^ T cells in MS. The search also targeted studies reporting on any aspects of T cells in MS. Studies that concentrated on animal models or individuals with other diseases were excluded from the review.

### 2.4. Study Selection

Each study selected for inclusion in this systematic review was independently screened on the basis of the title, abstract content, and full text. No standard assessment tool was utilized to evaluate the risk of bias or the quality of the basic research articles.

### 2.5. Ethical Approval

No ethical approval was required for this systematic review, as openly available published material was analyzed, and there was no involvement of human participants.

## 3. Results

A standard Google search using the selected keywords yielded 113 sources. The data were then screened for duplicates, thus decreasing the number of sources from 113 to 73. These sources were further checked, and 13 articles with abstracts or titles that did not conform to the current research question were removed. The selected articles reported on clinical trials and cross-sectional studies carried out within the last ten years that delved into the occurrence and role of CD3^+^CD20^+^ T cells in MS patients. The 15 most important studies included are given in Table 1.

The key findings highlighted in Table 2 offer sufficient insight into the occurrence and role of CD3^+^CD20^+^ T cells in MS patients. In particular, these cells are present in the CSF, and inflammation has possible implications for their population. Furthermore, CD3^+^CD20^+^ T cells may play a role in the general response of T cells to medications such as rituximab. These concepts can improve our understanding of the occurrence of CD3^+^CD20^+^ T cells and their clinical role in treatment interventions.

## 4. Discussion

### 4.1. CD3^+^CD20^+^ T Cells in CSF

CSF has been identified as a key component for understanding disease evolution in patients with MS [9]. Numerous findings have revealed the occurrence of intracellular immunoglobin (Ig) synthesis in more than 90% of MS patients [10]. Furthermore, clonally expanded B and T cells accumulate in MS lesions and in the CNS of patients with MS [11]. B cells tend to be present in the CSF of individuals with reported central nervous system (CNS) inflammation and are rarely present in the absence of such conditions [12]. T and B cells together often account for at least 30% of CSF cells in people with acute or chronic inflammatory neurological diseases [13]. As such, the role of T and B cells in CNS inflammation and in the progression of MS should be explored.

Recent research has shown that CD20^+^ T cells in the CNS are involved in inflammatory reactions in both patients with relapsing–remitting MS (RRMS) and those with progressive MS [14]. Therefore, ectopic follicular B cells are crucial for the pathogenetic response that drives disease progression. A previous study demonstrated significant increases in the proportions of B and T cells in the CSF of patients with RRMS [15,16,17,18]. The main conclusion from an assessment of previous studies is that the CSF count of T cells, particularly that of CD8^+^ T cells, correlates with MS diagnosis [19]. Specifically, the concentration of B and T cells is increased in the CSF of patients with RRMS [20,21,22,23]. This increase in the abundance of B- and T-cell subsets in MS patients is prompted by an increase in the IgG index, demonstrating the correlation between CSF and MS [24]. Indeed, CD3^+^CD20^+^ T cells occur at the same frequency as B cells and are mostly associated with enriched CD8^+^ T cells [25].

### 4.2. Occurrence of CD3^+^CD20^+^ T Cells in Progressive and Relapsing MS Patients

Studies have shown an increase in CD20^+^ T cells in the blood of patients with primary-progressive MS (PPMS) [26]. Therefore, the possible role of these cells in PPMS pathogenesis was investigated through flow cytometry, revealing an increased frequency of both CD4 and CD8 T cells in PPMS patients [27]. A previous investigation also confirmed the increased presence of CD20^+^ T cells within the CD4^+^ and CD8^+^ compartments of the CSF in patients with PPMS [28]. RRMS causes recurring episodes of inflammation and nerve damage, whereas PPMS progresses more gradually [29,30]. Increased levels of CD20^+^ T cells have been found in the blood of patients with RRMS [31]; these T cells are proinflammatory and exhibit increased reactivity toward myelin antigens. Furthermore, the prevalence of CD20^+^ T cells in the CSF of RRMS patients is associated with demyelination and disease severity [32].

Recent studies have also shown that CD20^+^ T cells are enriched in white matter lesions of patients with progressive MS, suggesting that these cells play a role in PPMS pathogenesis [33]. Previous findings on the enhanced migratory potential of CD20^+^ T cells have demonstrated that the proportions of CD20^+^ helper (CD4^+^) and cytotoxic (CD8^+^) T cells within the general CD4^+^ and CD8^+^ T-cell populations are considerably greater in the CSF than in the peripheral blood of patients with RRMS [34]. Other studies have shown similar frequencies of CD20^+^ T and B cells in the CSF of RRMS patients [35]. Untreated RRMS patients have more CD20^+^ T cells than B cells [36]. Therefore, it is critical to note that the frequency of CD20^+^ T cells is much greater in patients with RRMS than in those with PPMS; moreover, in RRMS patients, these cells exhibit greater migratory capacity toward the CNS than do CD20^−^ T cells.

CD3^+^CD20^+^ T cells were first reported in the early 1990s, when small fractions of CD3^+^ T cells that co-expressed CD20 were detected in the peripheral blood of individuals with HIV infection [37]. Further research using CD20 monoclonal antibodies (mAbs) demonstrated the presence of circulating CD3^+^CD20^+^ T cells in healthy people and patients with T-acute lymphoblastic leukemia [38]. Recent studies have shown that this subpopulation pervades the bone marrow, thymus, CSF, secondary lymphatic organs, liver, and brain [39]. Accordingly, the origin of CD3^+^CD20^+^ T cells in humans is controversial; CD3^+^ T cells in cord blood are primarily attributed to a lack of CD20 expression, whereas the CD3^+^CD20^+^ T cells in adult blood arise via trogocytosis, a process that involves the transfer of HLA-DR to T cells.

One approach to understanding CD3^+^CD20^+^ T cells in MS patients involves exploring the presence of CD20-expressing T cells in primary and secondary lymphatic organs. Research has shown that all lymphocytes in human blood from healthy donors express CD20 [40] and that CD20-expressing T cells are present in the blood, adenoids, and bone marrow. Further studies have focused on detecting CD3^+^CD20^+^ T cells in the thymus of young children and in mature medullary thymocytes. CD3^+^CD20^+^ T cells were detected in the CSF in the absence and presence of CNS inflammation and were consistently detected in patients who did not exhibit pleocytosis. Furthermore, CD3^+^CD20^+^ T cells were observed among lymphocytes in CSF from MS patients. In the absence of inflammation, CD20^+^ T cells tend to be more abundant than CD20^+^ B cells in CSF.

Another critical concept to explore is the expression of perforin and serine-protease granzymes by CD3^+^CD20^+^ T cells from MS patients. Studies on the cytotoxic behavior of CD3^+^CD20^+^ T cells in progressive MS revealed an increase in CD4^+^ T cells in the peripheral blood of RRMS patients, as reflected by the ectopic expression of serine-protease granzyme [41]; this effect was particularly evident during natalizumab therapy. Therefore, CD3^+^CD20^+^ T cells can express cytotoxic factors such as perforin in progressive MS. Hence, further characterization of the surface and functional markers of CD3^+^CD20^+^ T cells will enable the development of new therapeutic strategies for progressive MS and approaches for assessing the pathophysiological effects of anti-CD20 therapies.

### 4.3. Role of CD3^+^CD20^+^ T Cells in the Immune Pathology of MS

Supporting evidence for the role of the immune system in the pathogenesis of MS has emerged from the assessment of active demyelination lesions [42], which revealed some heterogeneity among patients. Despite this lesion heterogeneity, infiltrating cells corresponding to CD3^+^ T cells and macrophages were evident in the demyelinating plaques [43,44]. Notably, plasma cells account for only a small fraction of total cells. Another key finding from a cytometric examination of T cells was confirmation that fewer CD4^+^ T cells than CD8^+^ T cells are present in the active lesions of individuals suffering from MS [45]. CD8^+^ T cells also exhibit a tissue-resident phenotype and effector memory.

The possible role of CD20^+^ T cells in demyelination in patients with PPMS was previously investigated by focusing on the association between these cells and myelin basic protein (MBP) in CSF. The results revealed a strong positive correlation between the percentage of CD8^+^CD20^+^ T cells in CSF and the concentration of MBP, but there was no such correlation for CD20^+^ T cells [46]. Studies have also been carried out on patients with PPMS who are undergoing treatment; the results revealed a positive correlation between the frequency of CD8^+^CD20^+^ T cells and white matter lesion volume. Interestingly, there were negative correlations between intracellular CD8^+^CD20^+^ T cells and CD4^+^CD20^+^ T cells and thalamus volume.

Many studies have demonstrated that T cells play an essential role in the immune pathology of MS. The significance of these cells in disease pathology is based on their strong association with genes that are important for helper T (Th) cell functions [46]. Furthermore, the relevance of T cells is based on the idea that, in MS models, the adoptive transfer of myelin-reactive CD4^+^ T cells elicits disease-like pathology. T cells are key contributors to MS-related autoimmune demyelination, and there is sufficient evidence for a critical role of CD8^+^ T cells in MS immune pathology. Previous research has confirmed the overrepresentation of oligoclonal CD8^+^ T cells in MS lesions and the presence of myelin-reactive CD8^+^ T cells in the peripheral blood of MS patients. Moreover, these CD8^+^ T cells outnumber myelin-reactive CD4^+^ T cells.

Another exciting piece of evidence is that CD8^+^ T cells that react to glial fibrillary acid protein tend to mediate experimental RRMS. With such evidence, there is no doubt that an increasing number of CD8^+^ cells among CD3^+^CD20^−^ T cells can be found in patients diagnosed with MS (just normal CD8-Tcells). Treatment with rituximab can significantly impact the CD8^+^ and CD4^+^ subsets of CD20-expressing T cells. However, there is little evidence suggesting the contribution of the CD8^+^ subset to autoimmunity in MS. A similar conclusion can also be drawn for CD3^+^CD20^−^ T cells regarding whether their depletion indicates the efficacy of CD20-targeting therapies in MS.

Leukocytes, mainly T and B lymphocytes, are crucial for explaining the pathogenesis of MS. Inflammation in the CNS selectively recruits autoreactive T cells, followed by autoantigen targeting in brain tissues. Furthermore, autologous myelin-reactive T cells are primed for CNS autoantigens in the periphery before crossing the brain [47]. Upon reaching the brain, T cells can activate microglia and macrophages to trigger local inflammation. Numerous studies have documented the population-based distribution of these lymphocytes, with CD8^+^ T cells occurring at the edge and CD4^+^ T cells occurring deep in the lesions. Additionally, CD8^+^ T cells are often found in acute lesions, demonstrating their disease stage-specific role [48]. Importantly, autoreactive lymphocytes migrating toward the brain reduce regulatory T-cell function, promoting an autoimmune response in MS.

MBP-specific CD8^+^ T cells have also been argued to exacerbate brain inflammation through an autoimmune-encephalitis-like effect that promotes the formation of intracerebral and intracerebellar lesions. The role of these cells in the pathogenesis of MS is supported by the finding of an increased number of myelin antigen-specific circulating CD8^+^CD20^+^ T memory cells in MS patients [49]. As such, CD20 expression on these T cells is correlated with the upregulation of activation markers and proinflammatory cytokines, indicating high pathogenic potential. This finding leads to the conclusion that the transfer of CD20^+^ T cells negatively impacts the integrity of brain tissue and exacerbates disease severity. Furthermore, the proportion of CD20^+^CD8^+^ T cells is significantly reduced after anti-CD20 treatment, indicating the therapeutic potential of depleting CD20^+^ T cells in MS.

### 4.4. Role of CD3^+^CD20^+^ T Cells in MS Treatment

Research has demonstrated that mAbs targeting CD20 are crucial for reducing MS relapses, mainly due to their ability to deplete B cells [50]. Further inquiry revealed that anti-CD20 mAbs target a subset of CD3^+^CD20^+^ T cells [23]. Therefore, targeting CD20 with depleting mAbs represents a critical and promising therapeutic intervention for MS. The therapeutic success of this approach is solely attributed to the depletion of CD20^+^ B cells since CD20 is considered a specific B-cell marker. As such, CD20 is expressed during the development of B cells and maturation of pre-B cells. Conflicting reports also indicate that CD20 is expressed by some T cells. These findings suggest the presence of CD3^+^CD20^+^ T cells in human primary and secondary lymphatic organs and CSF. Notably, CD3^+^CD20^+^ T cells play a crucial role in the response to immunomodulatory treatments in MS patients.

Current evidence has established that untreated MS patients and healthy individuals might have similar frequencies of CD3^+^CD20^+^ T cells in their blood [51]. A strong response from this T-cell population is often observed with disease-modifying treatments. One example stems from the analysis of the effects of medications such as alemtuzumab, natalizumab, fingolimod, and dimethyl fumarate on the blood cells, specifically the CD3^+^CD20^+^ T cells, of MS patients [52]. Previous studies revealed that natalizumab increases the absolute number of CD20-expressing T cells and the relative frequency of CD3^+^CD20^+^ T cells among all lymphocytes [53]. Other drugs, such as rituximab, reduce the frequency of these cells. Overall, these drugs were found to deplete CD3^+^CD20^+^ T cells in the peripheral blood. For untreated patients, CD20^+^ B cells have been reported to be more common than CD20+ T cells in the blood, indicating the underlying role of CD3^+^CD20^+^ T cells in the treatment of MS.

Several studies have demonstrated the effects of anti-CD20 therapies on the T-cell compartment. Treatment of MS with rituximab reduces the T-cell population in the CSF and the circulating proinflammatory Th1 and Th17 cell responses of CD4^+^ and CD8^+^ cells [54]. Four anti-CD20 mAbs, namely, rituximab, ocrelizumab, ofatumumab, and ublituximab, have been examined for the treatment of MS. Most of these therapies differ in their recognition of CD20, but all have been proven to mediate near-complete depletion of CD20^+^ B cells in peripheral blood. Studies have shown that very few B cells remain in the blood after CD20-targeted depletion, while B cells in lymphoid tissue may resist depleting therapies [55,56]. The application of rituximab tends to reduce CSF B cells and has been proposed as a frontline treatment aimed at directly targeting B cells in MS. Additionally, ocrelizumab reduces the relapse rate and has been recommended for clinical development for PPMS and RRMS.

Current evidence suggests that CD20 is expressed at low levels in a small subset of T cells and can be effectively depleted from the peripheral blood by rituximab. However, further work should be conducted to determine whether these cells contribute to immune pathology in MS.

For several CD20 antibodies, including rituximab and ublituximab, researchers have already shown that a monoclonal CD20 antibody therapy is able to deplete the CD3^+^CD20^+^ T cells [26,56]. Lovett-Racke et al. were able to show that, in MS patients treated with an anti-CD20 therapy with ublituximab, both CD8^+^CD20^+^ T cells and CD4^+^CD20^+^ T cells were depleted almost completely [56,57]. Furthermore, in the study by Lovett-Racke et al., over 95% of these T-cells were CD45RA+, suggesting that these cells may have had a naïve cell-type rather than an aggressive or pathogenic cell-type. This shows that, in MS, the occurrence of CD3^+^CD20^+^ T cells potentially is purely epi-phenomenal and possibly not relevant for the pathogenesis in these patients [56,57].

The anti-CD20 mAb ocrelizumab, which was approved by the FDA in 2017 for the treatment of MS, has also been the subject of significant research to determine the role of CD20-expressing T cells in this disease. The function of T cells in the pathophysiology of MS has attracted considerable attention, as anti-CD20 therapies have shown impressive results in reducing disease activity [58,59]. For example, phase I and II studies on rituximab showed a rapid and pronounced reduction in inflammatory brain lesions and clinical relapse in RRMS patients. These results prompted interest in other therapies, which led to the approval of ocrelizumab, a humanized anti-CD20 mAb, for the treatment of RRMS and PPMS. Ocrelizumab substantially decreases gadolinium-enhancing lesions. Phase II studies of this drug were reported to show decreased disease activity and progression rates in patients with PPMS and RRMS.

However, there have been doubts regarding the role of CD20^+^ T cells in the mechanism of action of ocrelizumab. Earlier studies established that rituximab depletes these cells in MS patients. However, ocrelizumab exerts different cytotoxic effects, and the binding site for ocrelizumab to CD20^+^ T cells is not the same as that for rituximab. As such, it was necessary to determine whether ocrelizumab efficiently depletes T cells [60]. Previous research confirmed the conclusion regarding predominant co-expression of CD8 by CD20^+^ T cells in the peripheral blood of MS patients [61]. Researchers have reported the striking result of the efficient depletion of CD20^+^ T cells, which represent highly activated T-cell subsets with proinflammatory capacity. These results also explain the compelling effectiveness of anti-CD20 therapies, including ocrelizumab.

Other research has focused on the application of DMF treatment for patients with PPMS. The results revealed decreases in the absolute number of T cells, CD8^+^CD20^+^ T cells, and CD4^+^CD20^+^ T cells upon treatment with DMF [62]. Additionally, the number of CD8^+^CD20^+^ T cells in the CSF of these patients increased, but no effect on CD4^+^CD20^+^ T cells in this compartment was reported. In both cases, the population of CD20^+^ T cells was not affected, indicating that CD20^+^ T cells play a role in the compartmentalized immune response of the CNS in progressive MS.

### 4.5. Take-Home Message

CD3 is a surface T-cell marker, whereas CD20 is a surface B-cell marker. Traditionally, CD20 and CD3 are expressed on different immune cells and are used to accurately distinguish between CD20^+^ B cells and CD3^+^ T cells.

CD3^+^CD20^+^ double-positive T cells are derived from trogocytosis and/or the co-expression of these markers. Trogocytosis refers to the transfer of CD20 from B cells to T cells during B/T-cell interactions. This process then creates a double-positive cell population. Therefore, the identification of CD3^+^CD20^+^ T cells is indicative of a previous B/T-cell interaction.

There is some diagnostic value in identifying CD3^+^CD20^+^ T cells, but it has low specificity, as we typically do not know which process led to the generation of these cells.

The situation is different when CD3^+^CD20^+^ T cells are found in the CSF of MS patients. In this case, we can conclude that a B/T-cell interaction occurred in the CNS and that this interaction led to the generation of the identified CD3^+^CD20^+^ T cells. Therefore, this mechanism and the identification of CD3^+^CD20^+^ T cells in CSF have some diagnostic value in the context of the early diagnosis of MS.

CD3^+^CD20^+^ T cells, a subpopulation of CD3^+^ T cells that additionally express CD20, make up ~3–5% of the CD3^+^ T-cell compartment in peripheral blood. In healthy individuals, CD3^+^CD20^+^ T cells are heterogeneous in that they contain a lower proportion of CD4^+^ cells but produce higher levels of IL-17A and/or IFN-γ than CD3^+^CD20^−^ T cells do. Recent studies have shown the pathogenic behavior of CD3^+^CD20^+^ T cells in autoimmune diseases. These pathogenic cells can be removed by rituximab, an anti-CD20 antibody.

### 4.6. Practical Use

This review focuses on the occurrence of CD3^+^CD20^+^ T cells in patients with MS. Several authors have documented the occurrence of CD3^+^CD20^+^ T cells in patients with long-term MS. The importance of these cells for the treatment of MS patients has yet to be investigated in sufficient detail, and further studies are therefore needed. The occurrence of CD3^+^CD20^+^ T cells may be an indicator of chronic disease. Although some authors describe CD3^+^CD20^+^ T cells as aggressive, it is possible that the presence of these cells is related to treatment.

## 5. Conclusions

This comprehensive analysis of the role and occurrence of CD3^+^CD20^+^ T cells in MS patients revealed several important findings. An examination of the literature emphasized the presence of these cells in various locations within the human body, such as the bone marrow, peripheral blood, CSF, and lymphatic organs. The results substantiate previous research demonstrating the occurrence of CD3^+^CD20^+^ T cells in patients with certain inflammatory conditions. The origin of CD3^+^CD20^+^ T cells in humans is still a contentious topic, although trogocytosis has been suggested to be the mechanism underlying their presence in adult blood. However, their presence in CSF, even in the absence of inflammation, indicates their key role in the pathogenesis of MS.

Notably, the findings regarding the therapeutic implications of using drugs that target CD20 for the treatment of MS are equally important. MAbs targeting CD20 are efficacious at reducing relapses in MS patients by depleting CD20^+^ B cells. In contrast, the current review highlights that these therapeutic antibodies also target a subset of CD3^+^CD20^+^ T cells, demonstrating their multifaceted role in the immunopathology of MS.

Anti-CD20 mAbs have emerged as essential treatments for patients with PPMS and RPMS. However, it is critical to recognize the implications of the differences in the structure and target epitopes of these treatment options and the associated depletion mechanism. Current antibodies, such as rituximab, have proven to be efficacious in controlling disease activity and progression. In addition, the responses of these unusual immune cells to disease-modifying medications such as rituximab, natalizumab, and alemtuzumab underscore their potential as biomarkers of therapeutic efficacy. However, immunomodulation in MS management is a complex issue given that some drugs tend to increase the population of CD20-expressing T cells, whereas others have a depleting effect.

In conclusion, this study of CD3^+^CD20^+^ T cells provides compelling evidence for their participation in treatment responses and the pathogenesis of MS. Future research should focus on elucidating the precise mechanism underlying the function and emergence of CD3^+^CD20^+^ T cells in MS. Furthermore, extensive studies should be conducted to determine the potential of CD3^+^CD20^+^ T cells as therapeutic targets. Longitudinal studies are also recommended to further elucidate the dynamics of CD3^+^CD20^+^ T-cell responses to different medications and subsequent disease progression. Furthermore, the role of CNS-resident myelin-specific CD8^+^CD20^+^ T cells as effector cells in the demyelination process of PPMS awaits further inquiry. Therefore, it is crucial to address these knowledge gaps to advance our understanding of MS pathophysiology, which will lead to the development of efficacious therapeutic interventions that improve patient outcomes.

## Figures and Tables

**Table 1:** The 15 most important included studies.

Study Name	Title	Year
Sabatino et al. [9].	CD20 therapy depletes activated myelin-specific CD8^+^ T cells in multiple sclerosis. Proceedings of the National Academy of Sciences.	2019
Gingele et al. [10].	Role of CD20^+^ T cells in multiple sclerosis: Implications for treatment with ocrelizumab.	2020
Schuh et al. [11].	Features of human CD3^+^ CD20^+^ T cells. The Journal of Immunology.	2019
Sellebjerg et al. [12].	Anti-CD20 monoclonal antibodies for relapsing and progressive multiple sclerosis. CNS drugs.	2020
Palanichamy et al. [13].	Rituximab efficiently depletes increased CD20-expressing T cells in multiple sclerosis patients.	2014
Von Essen et al. [14].	Proinflammatory CD20^+^ T cells in the pathogenesis of multiple sclerosis. Brain.	2019
Chen et al. [15].	CD3^+^ CD20^+^ T cells and their roles in human diseases. Human Immunology.	2019
Delgado et al. [16].	Key characteristics of anti-CD20 monoclonal antibodies and clinical implications for multiple sclerosis treatment.	2023
Cross et al. [17].	Rituximab reduces B cells and T cells in the cerebrospinal fluid of multiple sclerosis patients.	2016
Meinl and Hohlfeld [18].	CD20^+^ T cells as pathogenic players and therapeutic targets in MS.	2021
Boldrini et al. [19].	Cytotoxic profile of CD3^+^ CD20^+^ T cells in progressive multiple sclerosis. Multiple sclerosis and related disorders.	2021
Gingele et al. [20].	Ocrelizumab depletes CD20^+^ T cells in multiple sclerosis patients.	2018
Von Essen et al. [21].	Intrathecal CD8^+^ CD20^+^ T cells in primary progressive multiple sclerosis. Neurology: Neuroimmunology & Neuroinflammation.	2023
Howlett-Prieto et al. [22].	Anti-CD20 therapy corrects multiple sclerosis’s CD8 regulatory T-cell deficit.	2021
Shinoda et al. [23].	Differential effects of anti-CD20 therapy on CD4 and CD8 T cells and implication for CD20-expressing CD8 T cells in MS disease activity.	2023

**Table 2:** Summary of the findings of the 10 most important studies.

	Findings
1	CD3^+^CD20^+^ T cells pervade the bone marrow, thymus, and secondary lymphatic organs [24].
2	CD3^+^CD20^+^ T cells are found in the CSF of MS patients [25].
3	Anti-CD20 monoclonal antibodies selectively deplete CD20^+^ B and T cells, efficiently suppressing inflammatory disease activity [26].
4	CD20^+^ T cells, which are reduced during rituximab therapy, play a pathogenic role in MS treatment [27].
5	Monoclonal antibodies targeting CD20 reduce the number of relapses in MS [28].
6	Rituximab and ublituximab efficiently deplete the increased population of CD20-expressing T cells in MS [29].
7	There is an increased frequency of CD20^+^ T cells in inflammatory conditions like MS [30].
8	A strong response of the CD20 T-cell population is often observed in disease-modifying treatments [31].
9	The immunopathogenesis of MS is primarily driven by deregulated T cells [32].
10	Disease-modifying therapies for MS mitigate inflammation by suppressing the activity of peripheral lymphocytes [33].

## Data Availability

Data will be made available upon request.
